# Public Masking: An Urgent Need to Revise Global Policies to Protect against COVID-19

**DOI:** 10.4269/ajtmh.20-0305

**Published:** 2020-04-22

**Authors:** Maryam Keshtkar-Jahromi, Mark Sulkowski, Kourosh Holakouie-Naieni

**Affiliations:** 1 Division of Infectious Diseases, Johns Hopkins University School of Medicine, Baltimore, Maryland;; 2 Department of Epidemiology and Biostatistics, School of Public Health, Tehran University of Medical Sciences, Tehran, Iran

Novel severe acute respiratory syndrome-coronavirus-2 (SARS-CoV-2) is spreading fast around the world, with many uncertainties about routes of transmission, treatment, and prevention. Currently, the United States has the highest number of cases. No drugs or vaccines have yet proven to be effective, and non-vaccine preventative measures are the key in our fight against this transmissible virus with high mortality. The WHO recommends robust control measures to contain community spread of infection worldwide, but the virus is nonetheless spreading within communities, and we have had limited success in breaking the transmission chain. Although new evidence supports tremendous capacity of this virus to spread from asymptomatic individuals, messages about public masking remain conflicting. More clarification from international organizations is needed to optimally support efforts to prevent the spread of SARS-CoV-2.

We now know that a significant portion of coronavirus-infected individuals are asymptomatic, or even minimally symptomatic with nonspecific symptoms,^1–3^ and can spread the infection.^4,5^ In a study from China, nasopharyngeal viral loads from asymptomatic individuals were as high as those from symptomatic patients, suggesting the potential for a major role in community transmission.^6^ In an observational study in China, asymptomatic SARS-CoV-2–infected individuals were identified as sources of infection in 79% of confirmed cases.^7^ During the Diamond Princess cruise ship investigation, 712 of 3,711 individuals tested positive, whereas 331 (46.5%) of positive cases were asymptomatic at the time of testing. In follow-up, 17.9% of infected cases never developed symptoms.^8^ Based on this understanding of virus transmission, public health experts urged mass masking to prevent virus spread.^9^

As of the time of this writing (mid-April 2020), the WHO recommends against public masking for asymptomatic individuals,^10^ but the science around this topic has been rapidly advancing. Some countries (Austria, Czech Republic, China, Hong Kong, Israel, Italy, Japan, Mongolia, Singapore, South Korea, Taiwan, Turkey, and the United States) have taken varied steps to advocate universal masking as an additional step to reduce community transmission of SARS-CoV-2. The WHO has not endorsed this as a global policy yet, possibly because of lack of supply, limited evidence for effectiveness, potential noncompliance, and/or concerns regarding increased anxiety and stigmatization engendered by masking. Moreover, there is concern that public masking will limit the supply of essential masks for healthcare workers and others at the highest risk of infection. However, a resistance to mass masking seems inconsistent with our knowledge of the rate of asymptomatic infections and the risk of transmission from these individuals.

The U.S. CDC has recommended a policy of cloth face covering in public where social distancing is hard to maintain, but this policy has been practiced in many different ways.^11^ Following this recommendation, mandatory universal masking for all hospital visitors, patients, and healthcare workers has been proposed^12^ and implemented as a robust preventive strategy in many American healthcare facilities including Massachusetts General Hospital, the Johns Hopkins University and Health System, the University of Maryland Medical System, the University of Chicago Medical Center, the University of Illinois Hospital, the University of Rochester Medical Center, the University of California San Francisco, the University of Virginia Heath System, Rochester Regional Health, and St. Luke’s University Health Network. This list is growing fast.

Outside of healthcare settings, for many people, the motivation for wearing a mask is primarily for their own protection, which unintentionally leads to protecting their community. We consider both protection of the wearer and of the community to be important, but we believe that source control is most important. In a systematic review before the SARS-CoV-2 pandemic, wearing face masks reduced the odds of contracting acute respiratory infections by 6% among casual community contacts and by 19% among household contacts if both the infected and healthy individuals wore masks.^13^ Masks have been shown to be effective in reducing respiratory virus shedding from droplets and aerosols of symptomatic individuals infected with coronavirus, influenza virus, and rhinovirus.^14^ There is also laboratory evidence that homemade masks effectively stop droplets from infecting the wearer.^15^

Surgical masks are disposable and designed to protect healthcare workers, although surgeons used cloth masks to protect wounds from droplets many years ago. The barrier layer in cloth masks is usually two layers of cotton-woven fabric, compared with two to four layers of nonwoven polypropylene fabric with filtration holes in surgical masks. Effectiveness of cloth masks compared with surgical masks needs further evaluation because some clinical studies have raised concerns regarding their effectiveness.^16^ However, the wearing of cloth masks by asymptomatic individuals is justified by evidence that droplets and aerosols get trapped when they hit the weave of the fabric, with potentially better trapping than surgical masks. In any case, cloth masks have been adopted as an alternative when surgical masks are limited in supply.^17^ Moreover, cloth masks are cheap, washable, easy to make, and can be used by the general population without imposing extra cost to local governments while they are already overwhelmed by lack of resources.

While the SARS-CoV-2 pandemic rages, most nations are not receiving clear and concise instructions about public masking from public health authorities. We call on WHO and country-level public health leaders to urgently consider revising their policies on mass masking to facilitate implementation of appropriate interventions in communities around the world. Considering the close community contact in densely populated areas around the world and documented evidence of SARS-CoV-2 transmission from asymptomatic individuals, it is appropriate to consider masking as a robust tool to limit SARS-CoV-2 transmission. This is highly recommended in public places including public transportation, grocery stores, bakeries, pharmacies, hospitals, rehabilitation units, nursing homes, offices, places of religious worship, workplaces, or even crowded streets. In summary, laboratory data support both surgical and cloth masks, but real-world efficacy of cloth masks needs further evaluation. Cloth masks are affordable globally and might be the only option in some areas with limited resources. Based on our current level of evidence, we highly recommend mass masking around the world during the pandemic. Whereas surgical masks are the preferred recommendation for the general public, cloth masks should be considered as a substitute if supplies are limited or surgical masks are not available.
